# Plasma indole-3-aldehyde as a novel biomarker of acute kidney injury after cardiac surgery: a reanalysis using prospective metabolomic data

**DOI:** 10.1186/s12871-023-02330-7

**Published:** 2023-11-07

**Authors:** Linhui Hu, Yunpeng Bai, Changchun Lai, Leitong Mo, Ying Li, Xinyi Jiang, Wang Xu, Yuemei He, Xinjuan Zhou, Chunbo Chen

**Affiliations:** 1 Department of Critical Care Medicine, Maoming People’s Hospital, No. 101 Weimin Road, Maoming, 525000 Guangdong Province China; 2 Center of Scientific Research, Maoming People’s Hospital, No. 101 Weimin Road, Maoming, 525000 Guangdong Province China; 3 Department of Clinical Laboratory, Maoming People’s Hospital, No. 101 Weimin Road, Maoming, 525000 Guangdong Province China; 4 Department of Coronary Care Unit, Maoming People’s Hospital, No. 101 Weimin Road, Maoming, 525000 Guangdong Province China; 5 Department of Intensive Care Unit of Cardiovascular Surgery, Guangdong Provincial People’s Hospital, Guangdong Cardiovascular Institute, Guangdong Academy of Medical Sciences, No. 106 Zhongshan Er Road, Guangzhou, 510080 Guangdong Province China; 6 School of Medicine, South China University of Technology, No. 382 Waihuan East Road, Guangzhou, 510006 Guangdong Province China; 7 The Second School of Clinical Medicine, Southern Medical University, No. 1023 Shatai South Road, Guangzhou, 510515 Guangdong Province China; 8 Department of Critical Care Medicine, Shenzhen People’s Hospital (The Second Clinical Medical College, Jinan University; The First Affiliated Hospital, Southern University of Science and Technology), Shenzhen, 518020 Guangdong China

**Keywords:** Indole-3-aldehyde, Biomarker, Predictor, Acute kidney injury, Cardiac surgery

## Abstract

**Background:**

Acute kidney injury (AKI) is a frequent complication of cardiac surgery that poses significant risks for both the development of chronic kidney diseases and mortality. Our previous study illustrated that heightened expression levels of faecal and plasma indole metabolites before the operation were associated with ischemic AKI. In this study, we aimed to validate the supposition that plasma indole-3-aldehyde (I3A) could serve as a predictive biomarker for AKI in patients undergoing cardiac surgery.

**Methods:**

This statistical reanalysis utilized AKI metabolomic data from patients scheduled for cardiac surgery between April 2022 and July 2022 in two tertiary hospitals. Faecal and blood samples were prospectively collected before surgery within 24 h, and variables related to the preoperative, intraoperative, and postoperative periods were recorded. AKI diagnosis was based on the Kidney Disease Improving Global Outcomes criteria.

**Results:**

In this study, 55 patients who underwent cardiac surgery were analyzed, and 27 of them (49.1%) developed postoperative AKI. Before surgery, these patients had significantly higher levels of faecal indole metabolites, including skatole, trans-3-indoleacrylic acid, and 5-methoxyindoleacetic acid. The plasma I3A, clinical model that considered perioperative and intraoperative variables, and their combination had area under the receiver operating characteristic curve (ROC) values of 0.79 (95% CI 0.67–0.91), 0.78 (95% CI 0.66–0.90), and 0.84 (95% CI 0.74–0.94) for predicting AKI, respectively. Furthermore, by utilizing net reclassification improvement and integrated discrimination improvement, plasma I3A showed significant improvements in risk reclassification compared to the clinical model alone.

**Conclusions:**

The dysregulation of gut microbiota metabolism in patients scheduled for cardiac surgery can result in an increase in indoles from tryptophan metabolism, which may be associated with postoperative acute kidney injury (AKI). This suggests that indoles may serve as a predictive biomarker for AKI in patients undergoing cardiac surgery.

**Supplementary Information:**

The online version contains supplementary material available at 10.1186/s12871-023-02330-7.

## Background

Millions of cardiac surgeries are performed annually throughout the world. The incidence of acute kidney injury (AKI) in patients undergoing cardiac surgery is high, ranging from 25 to 51% [1]. Evidence indicates that patients with AKI are more likely to suffer worsening clinical outcomes, including protracted hospitalisation [2], the need for renal replacement therapy (RRT) [3], the progression of new chronic kidney diseases (CKD) [4], and increased risk of mortality [5]. However, diagnosing AKI based on the rise in the level of serum creatinine (sCr) and urine output (UO) has fallen behind diagnosing it based on the initial injury of the kidney [6]. Under this circumstance, it is important to explore the novel markers for the early detection of AKI before a measurable rise in sCr, which can afford a critical time window for clinicians to stop or even reverse the progressing kidney injury. Although large numbers of biomarkers have been identified to detect AKI so far, including serum cystatin C (sCysC) [7, 8], urinary N-acetyl-beta-D-glucosaminidase (uNAG) [9], urinary matrix metalloproteinase-7 (uMMP-7) [10], and urinary angiotensinogen (uAGT) [11, 12], the search for an optimal and cost-effective combination scheme continues to be a prominent endeavor in the scientific community.

The gut microbiota plays an important role in the production of numerous compounds, influencing the host and the pathophysiological processes of a variety of diseases [13, 14], such as ischemic AKI [15, 16]. The bidirectional crosstalk between intestinal microbiota and the kidney has been confirmed through basic and clinical research involving the production of soluble factors and enterogenous uremic toxins [17]. Indoles are the most abundant tryptophan metabolites *via* the gut microbiota, which play crucial roles in the kidney system by activating the aryl hydrocarbon receptor (AHR) signaling pathway [18]. A recent study revealed that faecal indole contents and gut microbiota were different between patients with CKD and healthy control, which may provide gut symbiosis for patients with CKD as an additional treatment strategy [19]. However, only little is known about the correlation and influence of indole metabolites on the pathophysiological characteristics in AKI patients undergoing cardiac surgery.

In our previous study [20], we employed metabolomics analysis or statistical analysis to identify differential metabolites and altered metabolic pathways associated with CSA-AKI. We analyzed nearly 1000 faecal metabolites using high-resolution mass spectrometry (MS) and bioinformatics, and identified 49 differential metabolites with high confidence levels, which have potential diagnostic indicators and perform important biological functions. Additionally, we quantified and identified 188 plasma metabolites using tandem MS and found 34 differential plasma metabolites between the CSA-AKI and Non-AKI groups by using univariate statistical analysis. Notably, our research highlighted the significant role of indole metabolites, particularly I3A, in effectively distinguishing between the two groups. In the present study, the concerned indole metabolites were selected for clinical statistical analysis, which can verify the hypothesis that elevated indole metabolites before the operation is associated with ischemia AKI.

## Methods

### Participants and sample measurement

This prospective observational study was approved by the Ethics Committee of Maoming People’s Hospital (No. PJ2020MI-021-01) and conducted according to the Declaration of Helsinki at the Guangdong Provincial People’s Hospital and Maoming People’s Hospital. The study protocol complied faithfully with the Strengthening the Reporting of Observational Studies in Epidemiology [21] and the Standards for Reporting Diagnostic Accuracy criteria [22]. All participants provided their written informed consent. Eligible participants were those undergoing cardiac surgery at the cardiovascular surgical department from April 2022 to July 2022. The exclusion criteria for reanalysis were preexisting advanced CKD (end-stage kidney disease, kidney transplantation), age of > 80 years, a preexisting malignant tumour and refusal to consent. To assess the ability to predict AKI, patients who had been exposed to AKI from a prior operation were also excluded.

The occurrence of AKI based on Kidney Disease Improving Global Outcomes (KDIGO) criteria within 1 week of the cardiac surgery was defined as the main outcome [23]. As UO criteria may be affected by administrating diuretics or obesity, we adopted sCr to diagnose AKI. Other outcomes included the length of the intensive care unit (ICU) and hospital stay. Stool and blood samples were collected before the cardiac surgery and were then transported to the BGITech Company with dry ice for metabolomics experiments. Untargeted metabolomics was applied to stool samples, while the blood samples were assessed through targeted metabolomics, especially for plasma indole-3-aldehyde (I3A).

### Clinical data collection

Once the patients were accepted to the cardiovascular surgical department, their clinical data were collected. Preoperative variables, including demographics (i.e., age, height, weight, body mass index [BMI], gender and smoking), preexisting diseases (i.e., hypertension and diabetes mellitus), preoperative characteristics (i.e., Na+, K+, haemoglobin, red blood cell-specific volume, white blood cell [WBC], platelets [PLT], bilirubin, blood urea nitrogen [BUN], admission heart rate, admission respiratory rate, admission mean arterial pressure [MAP], NewYork Heart Association [NYHA] heart function) were recorded. Moreover, the preoperative baseline sCr was considered a preoperative renal function. Intraoperative data containing cardiopulmonary bypass time, operation time and blood loss were noted. The postoperative Acute Physiology and Chronic Health Evaluation (APACHE) II score, which was used to estimate the overall condition of a patient, was evaluated immediately after recovery from anaesthesia. The prognosis variables were also documented, which comprised the length of ICU and hospital stay. For the baseline sCr, we used the sCr at the time of hospital admission, the last available sCr within the last 3 months, or an estimated sCr as per the KDIGO guideline in patients with no prior information about their prior kidney function [24].

### Statistical analyses

The Shapiro–Wilk test was applied for continuous variables to determine the normal distribution. Student’s *t*-test was conducted for normally distributed data (presented as mean and standard deviation), while the Wilcoxon–Mann–Whitney U-test was used for skewed distributed data (presented as the median and interquartile range). Descriptive statistics for categorical variables were reported as frequency (percentage) and were compared using the Pearson χ^2^ test or Fisher’s exact test, as deemed appropriate. Using Fisher’s exact test or the Wilcoxon rank-sum test, the patient characteristics between AKI and Non-AKI were compared. The difference of the average of log2 peak areas concerning faecal indole metabolites or the concentrations of plasma I3A between the groups was calculated by Student’s *t*-test. Using simple logistic regression (SLR) models, the predictive performance of biomarkers was assessed. The SLR results were presented by the area under the receiver operating characteristic (ROC) curves (AUCs) and the corresponding 95% confidence intervals (CIs).

We calculated the C-index to assess the accuracy of predicting AKI and net reclassification improvement (NRI) and integrated discrimination improvement (IDI) to detect whether predicting accuracy could improve after the addition of plasma I3A into a baseline model with established risk factors. The established risk factors and clinical model were as follows: age, APACHE II score, haemoglobin and cardiopulmonary bypass time. The C-index was defined as the ROC between individual predictive probabilities for AKI and the incidence of AKI, and it was compared with both the baseline model (established risk factors only) and the enriched models (baseline model plus I3A). The NRI acted as a relative indicator of how many patients showed an improvement in the predicted probability of AKI, whereas the IDI indicated the average improvement in the predicted probability of AKI after adding the variables into the baseline model.

The statistical analysis was conducted with Python 3.9 using the SciPy 1.9.3 module, fundamental algorithms for scientific computing in python [25], and visualised with the Matplotlib 3.3.4 module [26]. P < 0.05 was considered to indicate statistical significance.

## Results

### Cohort description and baseline characteristics of the patients

A total of 55 patients with cardiac surgery were enrolled in the study after obtaining their written informed consent before the cardiac surgery, which included 47 patients from the Guangdong Provincial People’s Hospital and 8 patients from the Maoming People’s Hospital. The faecal and plasma samples were collected before cardiac surgery. With reference to the 2012 KDIGO clinical practise guideline, 27 patients (49.1%) were defined as AKI patients based on sCr elevation, and the other 28 Non-AKI patients served as the control. The basic information of patients, including age, gender, preoperative indicators and intraoperative indicators, is presented in Table 1. The patients with AKI were older than the Non-AKI patients. Moreover, prolonged operations and cardiopulmonary bypass could have increased the incidence of AKI. Poor renal function was also observed in patients with AKI, who had higher APACHE II scores at the time of ICU admission and a higher rate of CRRT during the longer hospital stay.

**Table 1:** Baseline characteristics and perioperative variables

Characteristics	AKI (n = 27)	Non-AKI (n = 28)	*P* Value
Age, years	64 (51, 67)	55 (45, 61)	0.009
Weight, kg	60.0 (56.0,69.0)	59.5 (52.8, 69.6)	0.555
Height, cm	165.0 (160.0, 170.0)	165.7 (160.5, 170.0)	0.742
Gender (male), n(%)	19 (70.4)	17 (60.7)	0.452
Diabetes, n(%)	1 (3.7)	1 (3.6)	1.000
Hypertension, n(%)	9 (33.3)	8 (28.6)	0.702
Smoking, n(%)	4 (14.8)	4 (14.3)	1.000
Admission heart rate, beat/min	79 (75, 85)	80 (75, 86)	0.378
Admission respiratory rate, breaths/min	20 (19, 20)	20 (20, 20)	0.057
Admission MAP, mmHg	99 (17)	103 (11)	0.402
Hospital stays, days	26 (18,30)	17 (14, 26)	0.033
ICU hospital stays, days	3 (2, 9)	3 (2, 4)	0.260
APACHE II score	7 (6, 9)	6 (4, 8)	0.035
NYHA heart function ≥ III, n(%)	8 (70.4)	17 (60.7)	0.452
CRRT, n(%)	5 (18.5)	0 (0)	0.055
**Preoperative indicators**			
Hemoglobin, g/L	120.15 (21.67)	132.64 (21.5)	0.036
WBC, 10^9^/L	7.17 (6.07, 8.55)	6.76 (5.26, 8.18)	0.213
PLT, 10^9^/L	216.41 (59.65)	243.89 (53.20)	0.077
Bilirubin, µmol/L	14.86 (9.70, 17.5)	12.65 (8.93, 19.08)	0.794
BUN, mmol/L	6.63 (5.78, 9.23)	6.43 (5.71, 8.22)	0.522
Creatinine, µmol/L	90.1 (69.1,125.0)	75.8 (60.9, 94.7)	0.042
eGFR, ml/min/1.73m^2^	92.7 (61.7, 114.6)	113.7 (78.6, 136.6)	0.0
**Intraoperative indicators**			
Cardiopulmonary bypass time, min	198 (151, 274)	143 (117, 209)	0.011
Operation time, min	400 (275, 515)	292 (223, 395)	0.019
Blood loss, ml	300 (200, 400)	200 (200, 300)	0.672

Results are presented as proportion for categorical variables, median (interquartile range) for continuous variables. BMI: body mass index, MAP: mean arterial pressure, CRRT: continuous renal replacement therapy, eGFR: estimated glomerular filtration rate, WBC: white blood cell, PLT: platelets, BUN: blood urea nitrogen

### Differential faecal indole metabolites as a predictor for the development of AKI

**Fig. 1:**
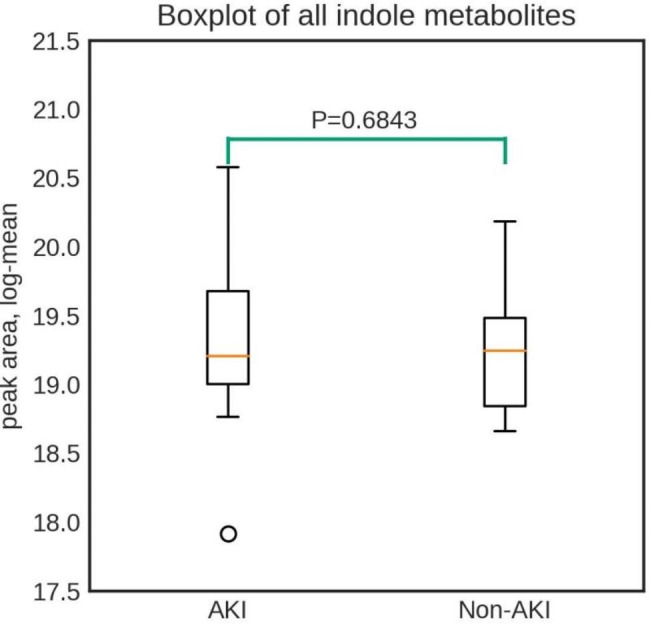
Boxplot illustrating the analysis of indole metabolites with a high confidence level in fecal samples prior to cardiac surgery. AKI, acute kidney injury

The faecal samples were analysed through untargeted metabolomics by high-resolution mass spectrometry, and all metabolites were annotated with five confidence levels as reported previously [27, 28]. In total, 23 indole metabolites with a high confidence level (Level 1–2) were employed in the comparative analysis between the AKI and Non-AKI groups (Table S1). After the log2 logarithmic transformation of the peak area, the average data were used to plot the box diagram. Figure 1 depicts that the contents of indole metaboles in the AKI group were higher than those in the Non-AKI group, albeit there was no significant difference between the two groups (*P* = 0.6978).

**Fig. 2:**
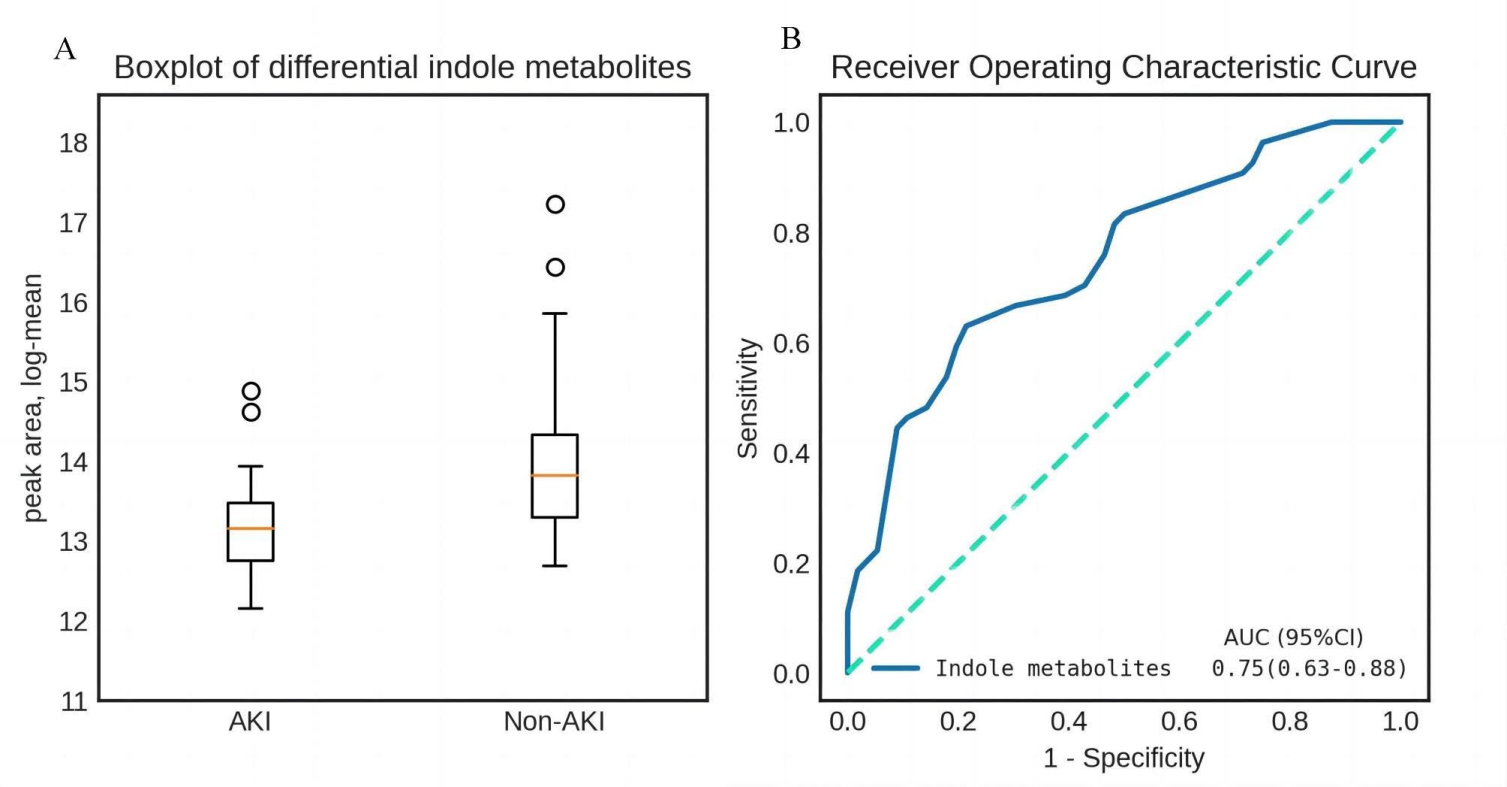
Differential analysis of indole metabolites in fecal samples prior to cardiac surgery. a Boxplot showing differential indole metabolites in AKI and Non-AKI. b ROC analysis for predicting AKI and Non-AKI. AKI: acute kidney injury, AUC: area under the ROC, ROC: receiver operating characteristic curve

The differential faecal metabolites related to AKI were screened out through univariate analysis, and 3 indole metabolites with a high confidence level (i.e., skatole, trans-3-indoleacrylic acid and 5-methoxyindoleacetic acid) were upregulated in the AKI group vs. the Non-AKI group. Using the abovementioned 3 differential indole metabolites, the *P* value of the boxplot was equal to 0.001 (Fig. 2A), and the AUC value was 0.75 (95% CI 0.63–0.88) in ROC analysis (Fig. 2B).

### Comparing the performance of plasma I3A with clinical mode for the development of AKI

**Fig. 3:**
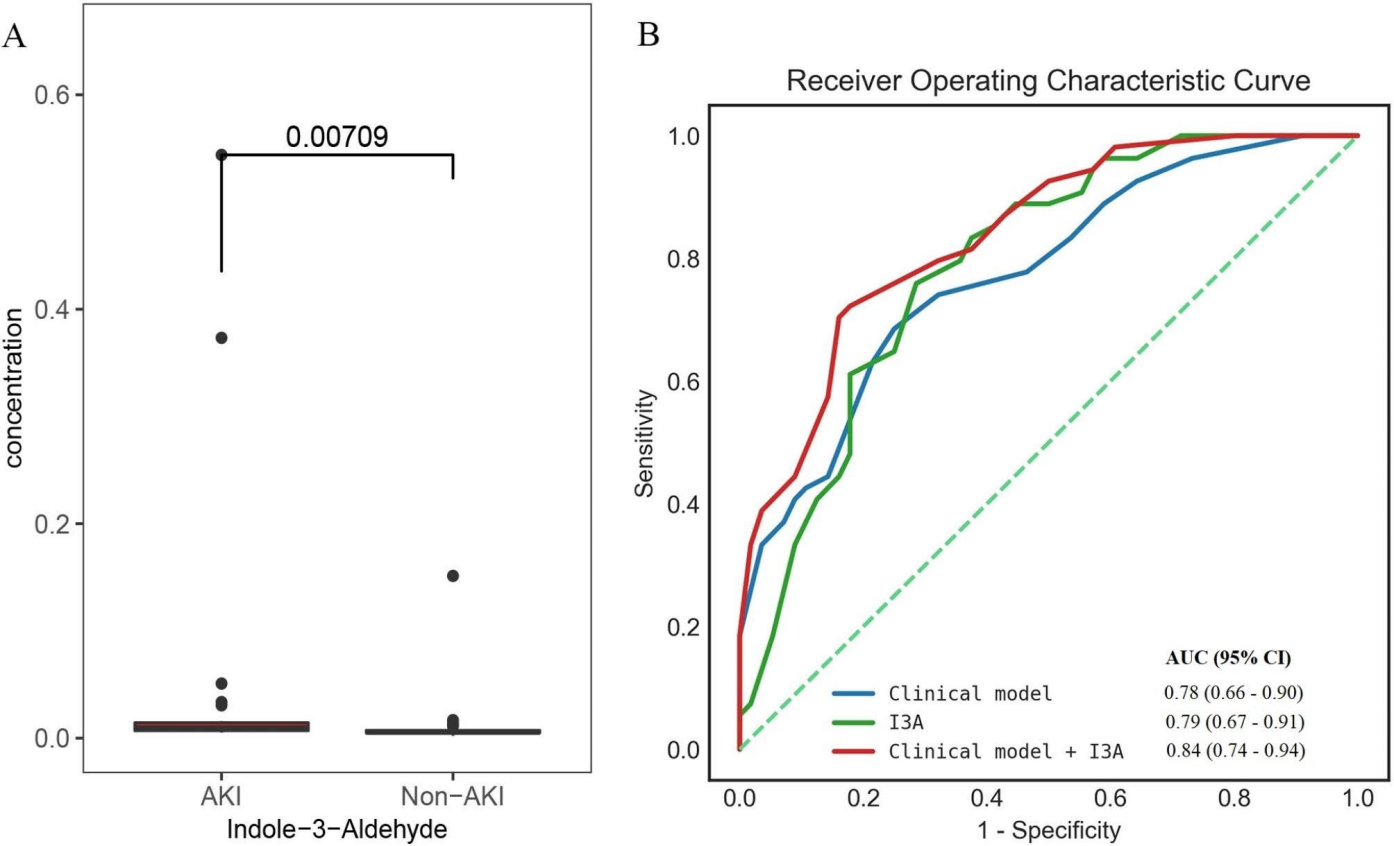
Analysis of I3A in plasma samples prior to cardiac surgery. a Boxplot showing differential I3A. b ROC analyses for predicting AKI and Non-AKI in all participants. The clinical model includes age, APACHE II score, hemoglobin, and cardiopulmonary bypass time, alongside I3A. AKI: acute kidney injury, APACHE II: acute physiology and chronic health evaluation II, AUC: area under the ROC, I3A: indole-3-aldehyde, ROC: receiver operating characteristic curve

The plasma samples were analysed by Metabolite Array using tandem mass spectrometry, which comprised amino acid, benzenoid, bile acid, carbohydrate, carnitine, fatty acid, indole, nucleoside, organic acid and phenylpropanoic acid. After univariate analysis, one indole metabolite (I3A) was screened out between the AKI and Non-AKI groups. Using a boxplot, Fig. 3A demonstrates that the plasma I3A values before surgery were obviously higher in patients with AKI during hospitalisation when compared with those in Non-AKI patients. For comparison, clinical mode, I3A and their combination were related to the incidence of AKI in patients undergoing cardiac surgery, with AUC values of 0.78 (95% CI 0.66–0.90), 0.79 (95% CI 0.67–0.91) and 0.84 (95% CI 0.74–0.94) in Fig. 3B, which means that plasma I3A may act as a predictive AKI biomarker after cardiac surgery. The clinical model comprised age, APACHE II score, haemoglobin and cardiopulmonary bypass time. Furthermore, when compared to only the clinical model, the addition of plasma I3A to the clinical risk model could significantly improve the risk reclassification (Table 2).

**Table 2:** Risk reclassification of postoperative AKI in cardiac surgery

Outcomes		Category-Free	*P* Value	Category-Free NRI				IDI (95% CI)	*P* Value
		NRI (95% CI)		With Event	*P* value	Without Event	*P* value		
AKI									
	Clinical risk factors	Referent						Referent	
	Clinical risk factors plus I3A	0.83 (0.36–1.29)	0.0005	19%	0.3275	64%	0.0000	0.09 (0.02–0.16)	0.0132

The established risk factors included age, APACHE II score, hemoglobin and cardiopulmonary bypass time. AKI, acute kidney injury; APACHE, Acute Physiology and Chronic Health Evaluation; CI, confidence interval; IDI, integrated discrimination improvement; NRI, net reclassification improvement

## Discussion

This is the first study to demonstrate that the elevation of plasma I3A is an independent predictor for patients with cardiac surgery associated-AKI. The present results indicates that plasma I3A is a moderate predictor for AKI in patients undergoing cardiac surgery, which demonstrated almost a consistent performance (AUC = 0.79) with the clinical model (AUC = 0.78) in predicting AKI. The clinical model was developed using multivariable logistic regression analysis and ultimately incorporated age, APACHE II score, pre-surgery hemoglobin levels, and cardiopulmonary bypass time.

As demonstrated by the AUCs, the addition of plasma I3A to the clinical model could further increase the area of AUC from 0.78 (clinical mode only) to 0.87 (the combination of clinical mode and I3A). When measured by category-free NRI (value = 0.83) and IDI (value = 0.09), the risk reclassification was markedly improved by adding plasma I3A to the clinical model (*P* < 0.05). Thus, preoperative plasma I3A can be considered an independent predictor for cardiac surgery-associated-AKI, which may aid in the identification of patients in whom preventive treatment strategies can be effective [29].

The current study is a reanalysis of data from a previous study [20] that investigated the relationship between gut microbiota metabolism and acute kidney injury (AKI) following cardiac surgery. However, this study specifically focuses on evaluating the predictive value of plasma indole-3-aldehyde (I3A) as a biomarker for AKI. The analysis included 55 patients, of whom 27 developed postoperative AKI. The study revealed significantly elevated levels of specific faecal indole metabolites in patients who experienced AKI, indicating an imbalance in gut microbiota metabolism. Furthermore, the study demonstrated that plasma I3A, in combination with perioperative and intraoperative variables, exhibited strong predictive performance for AKI. Importantly, the addition of plasma I3A to the clinical model significantly improved risk reclassification for AKI. These findings provide additional evidence supporting the involvement of dysregulated gut microbiota metabolism and indole production in the development of postoperative AKI. The study underscores the significance of metabolomic analysis and highlights the potential clinical utility of plasma I3A as a diagnostic tool for identifying individuals at risk of AKI after cardiac surgery. Overall, this study contributes valuable insights by validating plasma I3A as a promising predictive biomarker for AKI in cardiac surgery patients.

Notably, opposite to the I3A, the pre-surgery creatine did not enter the clinical model despite there was significantly higher preoperative creatinine levels in the AKI group compared to the Non-AKI group. This outcome may be attributed to the lack of observed correlation in improving the model when patients with advanced chronic kidney diseases (CKD) were excluded. Another possible contributory factor could be the limitation of creatinine in accurately reflecting the true renal function reserve, as it is influenced by variables such as muscle mass and protein intake, which can impact its reliability as a measure of renal function. Moreover, the elevated creatinine in the AKI group may be attributed to older age, as also indicated in Table 1, resulting in lower creatinine clearance. It is worth noting that although there were differences in creatinine values, they still remained within the normal range, and no significant difference was observed in blood urea nitrogen (BUN). Unlike creatinine, eGFR takes into account serum creatinine level, age, and gender, making it a more reliable indicator of renal function. Our findings indicate that eGFR did not remain in the model as well. However, given the well-established link between CKD and AKI after cardiac surgery, we aim to enhance our understanding of how CKD impacts our model results and evaluate the added classifying performance of I3a in CKD or Non-CKD patients in further study with CKD incorporated as a screening variable.

APACHE II score was also a predictor in the clinical model free of the collinearity with creatine prior to surgery, which can be attributed to several factors. Firstly, these variables serve different purposes and capture different aspects of evaluation. Secondly, the utilization of distinct measurement methods for APACHE II score and creatinine may have diminished their correlation. Lastly, rigorous data preprocessing techniques and consideration of sample characteristics likely mitigated the potential presence of collinearity. In addition, it should be noted that the relatively small sample size in our study may introduce some variability in blood creatine levels. To address this potential imbalance between the groups, we are planning to conduct a prospective study with a significantly larger sample size. This will allow for more robust and reliable conclusions by minimizing the effects of any observed discrepancies.

Plasma I3A demonstrated the ability to discriminate subgroup, suggesting that it may detect kidney injury earlier than creatinine. It is worth noting that plasma indole metabolites have been shown to be correlated with CKD [30, 31] and atherosclerosis [29], leading to speculation that atherosclerosis rather than I3A might be the underlying risk factor for CSA-AKI. However, it is important to highlight that our study specifically excluded patients with CKD, thus eliminating the confounding effect of CKD. Moreover, multiple logistic regression analysis, which helps control for confounding factors and identify independent influencing factors, had been previously employed by Cason et al. [30]. They adjusted for traditional risk factors and identified plasma indoxyl as an independent risk factor for atherosclerosis. Similarly, in our study, we also adjusted for hypertension and diabetes, which are the two primary risk factors for atherosclerosis, and obtained consistent results, affirming I3A as an independent predictor for CSA-AKI development. These findings further support the notion that I3A serves as an independent predictor for CSA-AKI.

As for AUCs, both plasma I3A and clinical mode demonstrated fair prediction ability owing to their AUC values of 0.7–0.8, while the combination displayed good ability owing to the AUC value of 0.8–0.9 [31, 32]. In addition, the risk reclassification (as measured by category-free NRI and IDI) was significantly improved by adding plasma I3A to the clinical model; therefore, it was helpful to increase the discrimination by adding plasma I3A. All these findings affirm the notion that it was insufficient for clinicians to evaluate AKI based on a single biomarker. Instead, a multi-biomarker approach should be adopted in clinical practice [33]. However, the AUC was not markedly improved by adding plasma I3A to the clinical model according to the current data (*P* > 0.05), which indicated that we should continue to expand the samples and conduct a multi-centre prospective study to further verify the improvement of I3A to AUC.

As an important metabolite of tryptophan, indole compounds are under the direct or indirect control of the gut microbiota, which is involved in microbiota-host crosstalk across health and disease sectors [34]. I3A, produced by the indole-3-pyruvic acid pathway and aromatic amino acid aminotransferase *via* the gut microbiota [35], can activate AHR to enhance tumour malignancy and suppress anti-tumour immunity [36]. As molecular and animal studies on the mechanism of ischemia AKI in the signalling pathway relevant to plasma I3A remain inadequate, further studies are deemed necessary to identify the optimal mechanism of plasma I3A in ischemia AKI. Indoxyl sulphate, a representative uraemic toxin in the blood of patients with CKD, can increase the morbidity and mortality of the disease’s related complications; therefore, timely detection of its level may effectively prevent the progression of CKD and its related complications [37]. Therefore, the early detection of plasma I3A before cardiac surgery may assist clinicians in recognising patients who are more prone to suffer AKI to cure or reverse the sustaining kidney injury in a vital time window [38].

Our study offers several strengths. First, we measured the performance of plasma I3A preoperatively in patients undergoing cardiac surgery. Consequently, as the time nodes of initial injury in the kidney were accurate, the variations among patients due to the individual differences in preexisting but unclear initial kidney injury and the underlying comorbidities could be reduced. Second, the samples were prospectively gathered at different centres; therefore, the accuracy of internal verifiability could be improved. Third, the early detection of plasma I3A can facilitate clinicians to recognise patients at a high risk of developing postoperative AKI for the initiation of efficient intervention in a timely manner. Indeed, the performance of plasma I3A was not satisfactory in AUC. However, this study was the pilot exploratory research. We plan to further improve the experimental design to increase AUC, NRI and IDI in the subsequent confirmatory experiments.

Nevertheless, this study has several limitations. First, as UO was not used in the diagnosis of AKI, it may have led to the omission of a portion of the incidence of AKI [39]. However, the incidence of AKI in patients undergoing cardiac surgery was consistent with that reported previously [40]. Second, the study participants included those undergoing cardiac surgery in two centers, which represent patients from a few simple clinical settings. In the future, we plan to amplify our participants from multiple centres and departments across different causes of ischemic AKI, such as patients with decompensated heart failure and other surgeries [41].

## Conclusion

The dysregulation of gut microbiota metabolism in patients scheduled for cardiac surgery can result in an increase in indoles from tryptophan metabolism, which may be associated with postoperative acute kidney injury (AKI). This suggests that indoles may serve as a predictive biomarker for AKI in patients undergoing cardiac surgery.

### Electronic supplementary material

Below is the link to the electronic supplementary material.

Supplementary Material 1

## Data Availability

The datasets generated and analysed during the current study are available in the ProteomeXchange Consortium repository, https://www.iprox.cn/page/project.html?id =IPX0005153000.
